# A Case with a Pathological Course Resembling Ulcerative Colitis after Rectal Cancer Surgery with Diversion

**DOI:** 10.70352/scrj.cr.26-0074

**Published:** 2026-04-09

**Authors:** Tadaaki Shimizu, Masato Kitazawa, Atsuhiro Hirayama, Satoshi Nakamura, Yuta Yamamoto, Satoru Miyazaki, Nao Hondo, Masahiro Kataoka, Hirokazu Tanaka, Masatsugu Kuroiwa, Naoki Ishizaka, Naoya Yamamoto, Yuji Soejima

**Affiliations:** 1 Division of Gastroenterological, Hepato-Biliary-Pancreatic, Transplantation and Pediatric Surgery, Department of Surgery, Shinshu University School of Medicine, Matsumoto, Nagano, Japan; 2 Division of Gastroenterology, Department of Internal Medicine, Shinshu University School of Medicine, Matsumoto, Nagano, Japan

**Keywords:** ulcerative colitis, diversion colitis, rectal cancer surgery, anastomotic complication, diverting stoma

## Abstract

**INTRODUCTION:**

The development of *de novo* ulcerative colitis (UC) is rare after colorectal cancer surgery. In contrast, diversion colitis is a common inflammatory condition of the defunctionalized colon following stoma creation, often complicating postoperative diagnosis.

**CASE PRESENTATION:**

A 49-year-old man with advanced rectal cancer was treated with neoadjuvant chemotherapy followed by robot-assisted ultra-low anterior resection with diverting stoma. His postoperative course was complicated by an anastomotic leak. During the diversion period, he developed fever, diarrhea, and colonic inflammation that was initially diagnosed as diversion colitis and responded dramatically to systemic steroid therapy. Following stoma closure, recurrent anastomotic ulceration and diffuse colitis developed, again showing marked steroid responsiveness. Based on the overall clinical course, the patient was considered to have a clinical course resembling UC rather than diversion colitis. The patient achieved sustained remission with systemic steroids and mesalamine, with no recurrence of intestinal inflammation or cancer.

**CONCLUSIONS:**

This case underscores the challenges in distinguishing UC from diversion colitis in the postoperative setting. Recurrent anastomotic inflammation with pronounced steroid responsiveness should prompt consideration of underlying UC, even after colorectal cancer surgery.

## Abbreviations

CRP
: C-reactive protein
CTRX
: ceftriaxone
PSL
: prednisolone
UC
: ulcerative colitis

## INTRODUCTION

UC is a chronic inflammatory bowel disease characterized by persistent inflammation of the colonic mucosa along with an increased risk of developing colorectal cancer. Although UC may worsen in response to physical or psychological stress, including surgical stress, *de novo* development or initial manifestation of UC after colorectal cancer surgery is exceedingly rare, with only a limited number of cases reported.^1,2)^

Diversion colitis is a well-recognized, nonspecific inflammatory condition that occurs in the defunctionalized colon after stoma creation, most commonly following rectal cancer surgery. Inflammatory changes are observed in nearly all diverted colonic segments; however, most patients remain asymptomatic, and only approximately 30% develop clinical symptoms.^3,4)^ Furthermore, histopathological findings are often nonspecific, including crypt abscesses or granulomatous changes, making the differentiation of UC and other inflammatory bowel diseases particularly challenging in the postoperative setting.^5,6)^

Herein, we report a rare and clinically significant case with a clinical course resembling UC following low anterior resection for rectal cancer. Notably, the patient experienced 3 anastomosis-related complications—anastomotic leak in the immediate postoperative period, deep ulceration at the anastomotic site before the initiation of postoperative chemotherapy, and recurrent ulceration after stoma closure—that resulted in significant diagnostic and therapeutic challenges. Systemic steroid therapy was found to be effective in all episodes, leading to marked clinical improvement. This case highlights the importance of recognizing the inflammatory potential of the colonic mucosa before colorectal surgery and underscores the diagnostic challenges in distinguishing UC from diversion colitis in postoperative scenarios.

**Fig. 1:**
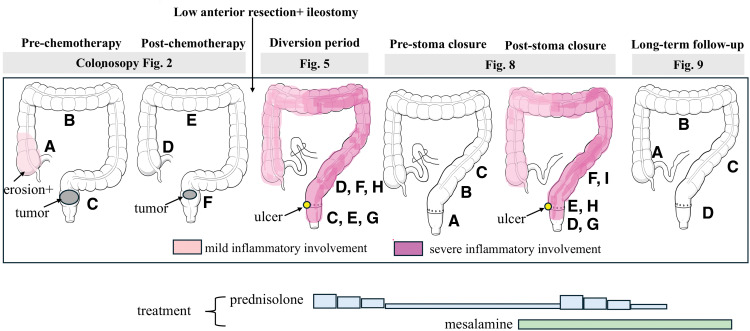
Distribution and temporal changes of colitis during the clinical course. Schematic illustration showing the anatomical distribution and severity of colonic inflammation at each stage of the clinical course. Mild inflammatory involvement is shown in light pink, whereas severe inflammatory involvement is shown in dark pink. During the diversion period, diffuse inflammation developed in the proximal colon with ulcer formation near the anastomotic site. After stoma closure, recurrent inflammation involved both the rectum and proximal colon. Long-term follow-up colonoscopy demonstrated complete resolution of inflammatory changes.

The distribution and temporal changes of colitis throughout the clinical course are summarized in **Fig. 1**.

## CASE PRESENTATION

A 49-year-old man was diagnosed with advanced rectal cancer, clinically staged as cT3N3 with lateral pelvic lymph node involvement and distant metastasis to the left supraclavicular lymph node (M1). Genetic analysis revealed a KRAS G13D mutation.

**Fig. 2:**
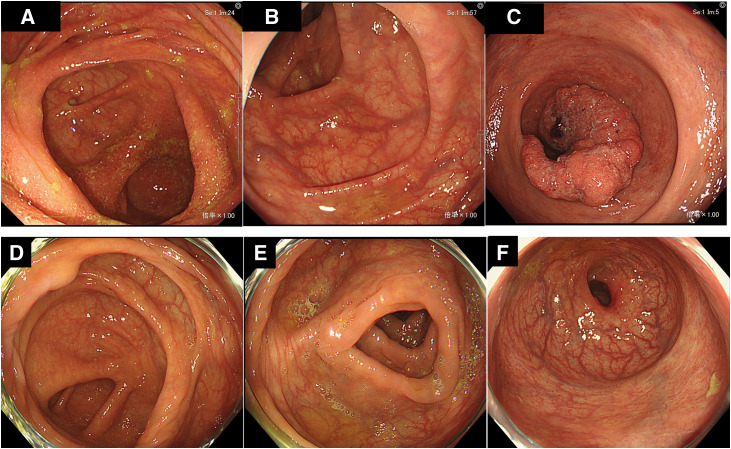
Colonoscopy findings before and after systemic chemotherapy. (**A**) Pre-chemotherapy colonoscopy revealed mucosal erosions in the cecum. (**B**) The transverse colon showed no remarkable findings before chemotherapy. (**C**) In the rectum (Rb), above the second Houston valve, a Type 2 tumor involving approximately two-thirds of the luminal circumference was identified. (**D**) Post-chemotherapy colonoscopy demonstrated resolution of the previously observed cecal mucosal erosions. (**E**) No inflammatory changes were observed in the transverse colon after chemotherapy. (**F**) The rectal tumor had almost completely regressed on post-chemotherapy endoscopic examination.

Pre-chemotherapy colonoscopy revealed mucosal erosions in the cecum (**Fig. 2A**) and no remarkable findings in the transverse colon (**Fig. 2B**). A Type 2 tumor involving approximately two-thirds of the luminal circumference was identified in the rectum (Rb) above the second Houston valve (**Fig. 2C**). The inflammatory changes observed in the cecum were considered nonspecific colitis, as no diffuse or continuous inflammatory findings suggestive of inflammatory bowel disease were observed.

The patient received systemic chemotherapy with FOLFOXIRI plus bevacizumab. The left supraclavicular lymph node metastasis showed a complete radiological response after six chemotherapy cycles. Post-chemotherapy colonoscopy revealed resolved cecal mucosal erosions (**Fig. 2D**), with no inflammatory changes in the transverse colon (**Fig. 2E**). Endoscopic examination revealed an almost complete regression of the rectal tumor (**Fig. 2F**).

**Fig. 3:**
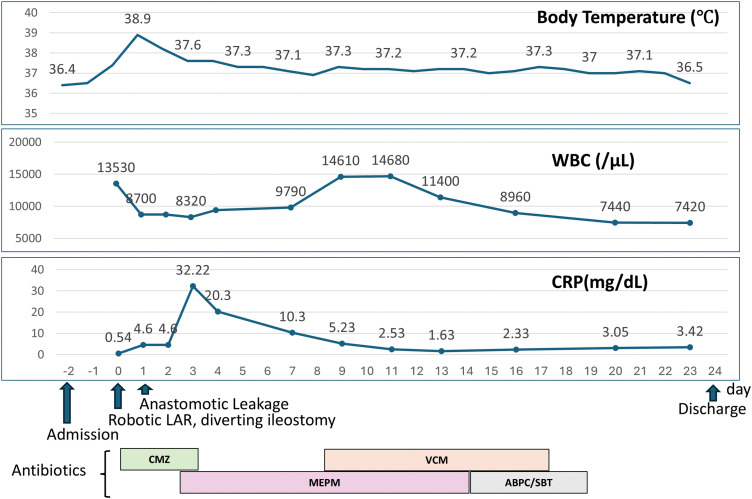
Postoperative clinical course after robot-assisted ultra-LAR. The figure summarizes the postoperative course, including changes in body temperature, WBC count, and CRP levels, along with the timeline of antibiotic therapy. On POD 1, feculent drainage from the surgical drain and a high-grade fever developed, and CT findings were consistent with anastomotic leak. The patient was managed conservatively with antibiotics and drainage. Despite persistent contaminated drainage and prolonged elevation of inflammatory markers, no severe abdominal pain was noted, and oral intake was maintained. The surgical drain was exchanged and left in place, and the patient was discharged on POD 24 with the drain in situ. ABPC/SBT, ampicillin/sulbactam; CMZ, cefmetazole; CRP, C-reactive protein; LAR, low anterior resection; MEPM, meropenem; VCM, vancomycin; WBC, white blood cell

Subsequently, robot-assisted ultra-low anterior resection with right lateral pelvic lymph node dissection was performed. Pathological examination revealed ypT3N0M0 disease, corresponding to Stage II. A diverting stoma was constructed at the time of surgery. **Figure 3** summarizes the postoperative course, including changes in body temperature, white blood cell count, CRP levels, and the timeline of antibiotic therapy. Feculent drainage from the surgical drain and a high-grade fever developed on the first POD. CT demonstrated findings consistent with anastomotic leak, and the patient was managed conservatively. Thereafter, despite adequate drainage, persistent fever and prolonged elevation of inflammatory markers were observed, without severe abdominal pain. Despite persistent contaminated drainage from the surgical drain, the patient was able to tolerate oral intake and exhibited no other significant clinical symptoms; therefore, the surgical drain was exchanged and left in place. The patient was discharged on POD 24 with the drain in situ.

**Fig. 4:**
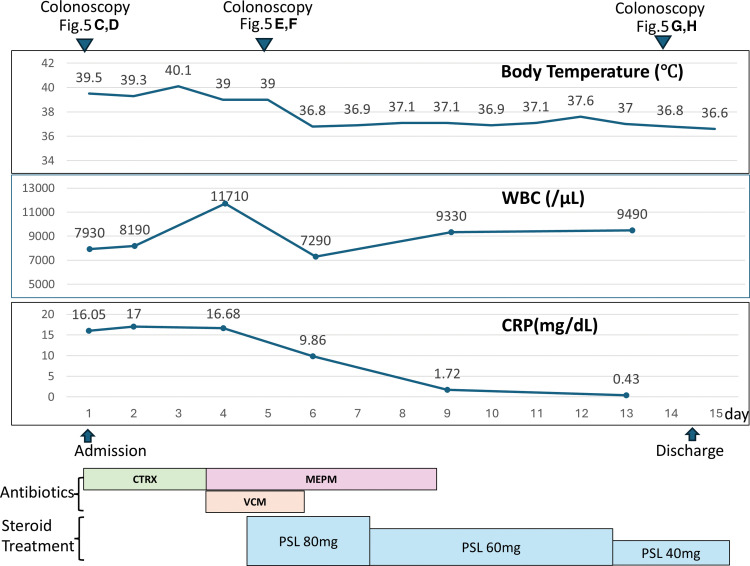
Clinical course during readmission after surgery. The figure illustrates changes in body temperature, WBC count, and CRP levels, along with the timeline of antibiotic and steroid therapy during readmission. Despite broad-spectrum antibiotic treatment, fever and inflammatory markers persisted. Intravenous corticosteroid therapy was subsequently initiated, leading to rapid improvement in clinical symptoms and inflammatory parameters, followed by gradual tapering of steroids and discharge. CRP, C-reactive protein; CTRX, ceftriaxone; MEPM, meropenem; PSL, prednisolone; VCM, vancomycin; WBC, white blood cell

**Fig. 5:**
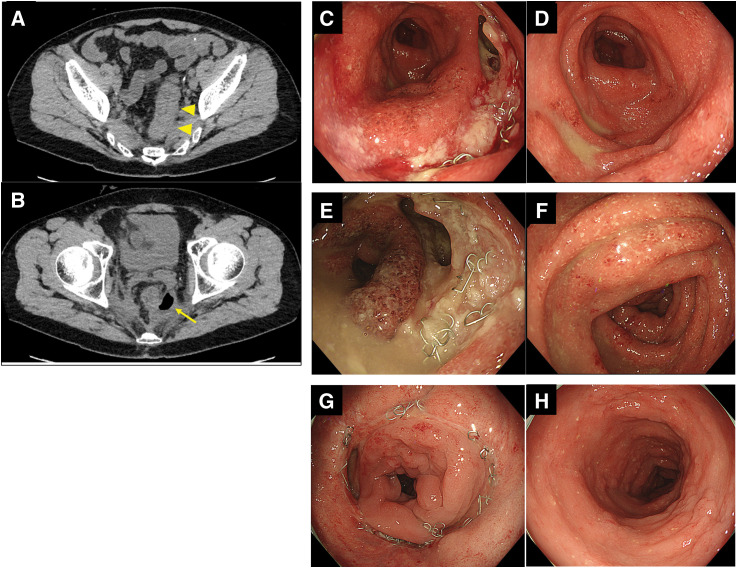
Imaging and endoscopic findings during readmission. (**A**) CT showing edematous wall thickening of the proximal bowel at the anastomotic site in the descending colon (arrowheads). (**B**) Fluid collection containing air adjacent to the anastomosis without evidence of free intraperitoneal air (arrow). (**C**) Colonoscopy on admission demonstrating a deep ulcer at the anastomotic site. (**D**) Diffuse erythema with purulent erosions in the proximal colon. (**E**) Repeat colonoscopy on Day 5 post-hospitalization showing progression of the anastomotic ulcer. (**F**) Worsening inflammatory changes in the proximal colon. (**G**, **H**) Follow-up colonoscopy 9 days after initiation of intravenous corticosteroid therapy demonstrating marked healing of the anastomotic ulcer and resolution of inflammatory changes.

**Fig. 6:**
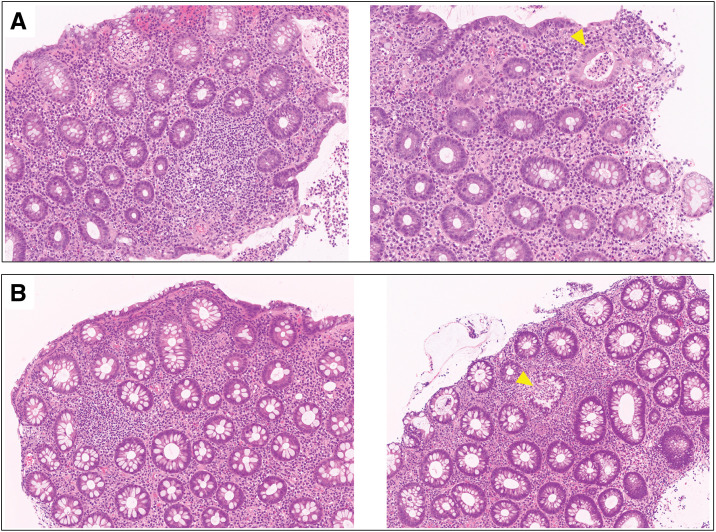
Histopathological findings of colonic biopsy specimens (hematoxylin and eosin staining). (**A**) Biopsy specimens obtained during readmission demonstrating chronic inflammation with dense inflammatory cell infiltration in the mucosal stroma, accompanied by cryptitis and crypt abscesses (arrowhead). (**B**) Biopsy specimens obtained during exacerbation after stoma closure showing similar histopathological features, including marked inflammatory cell infiltration, cryptitis, and crypt abscess formation (arrowhead).

More than 2 months after surgery, the patient was readmitted with mild lower abdominal pain, watery diarrhea, high-grade fever, and elevated inflammatory markers (**Fig. 4**). CT demonstrated edematous wall thickening of the proximal bowel at the anastomotic site, corresponding to the descending colon (**Fig. 5A**), along with a fluid collection containing air adjacent to the anastomosis, without evidence of free intraperitoneal air (**Fig. 5B**). Colonoscopy was performed on the same day; endoscopic examination revealed a deep ulcer at the anastomotic site (**Fig. 5C**) and diffuse erythema with purulent erosions at the proximal colonic mucosa (**Fig. 5D**). Broad-spectrum antibiotics were initiated owing to the possibility of infectious enterocolitis; however, fever and watery diarrhea persisted until Day 5 post-hospitalization. Although a diverting stoma had been constructed, the diarrhea consisted of fecal discharge from the anus. *Clostridioides difficile* antigen testing was negative, and stool cultures did not detect pathogenic bacteria, including enteropathogenic *Escherichia coli*. Cytomegalovirus infection was also considered in the differential diagnosis; however, histopathological examination of biopsy specimens did not reveal cytomegalovirus inclusion bodies. A repeat colonoscopy on Day 5 post-hospitalization demonstrated progressive ulceration at the anastomotic site (**Fig. 5E**), along with worsening inflammatory changes in the proximal colon (**Fig. 5F**). Histopathological examination of biopsy specimens revealed chronic inflammation with neutrophil infiltration in the mucosal stroma, along with cryptitis and crypt abscesses extending from the ascending colon to the Rb (**Fig. 6A**). A diagnosis of diversion colitis was made based on these findings, and intravenous corticosteroid therapy was initiated. Fever and diarrhea responded rapidly to the steroid therapy, reducing significantly within 1 day after therapy initiation. Follow-up colonoscopy performed 9 days after the initiation of steroid therapy demonstrated dramatic improvement of the anastomotic ulcer and resolution of the inflammatory changes in the proximal colon (**Fig. 5G** and **5H**). The patient was discharged 10 days after steroid initiation, and the steroid dose was gradually tapered to low-dose oral maintenance therapy.

While receiving oral PSL at doses ranging from 10 to 5 mg/day, the patient completed 6 cycles of postoperative FOLFOX chemotherapy. The PSL dose was subsequently tapered to 1 mg/day, and stoma closure was performed several months later under continued low-dose PSL.

**Fig. 7:**
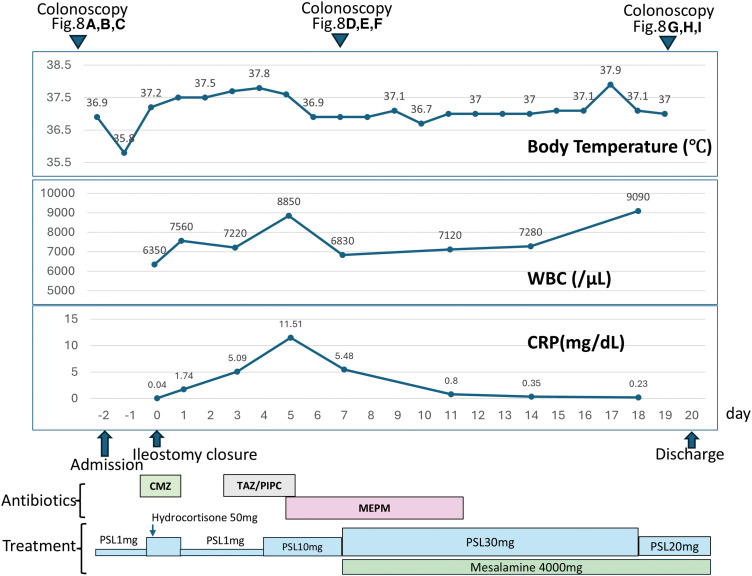
Perioperative clinical course of stoma closure. The figure summarizes changes in body temperature, WBC count, and CRP levels during the perioperative period of stoma closure, together with the timing of colonoscopy examinations, antibiotic therapy, and medical treatment. Despite perioperative steroid coverage and antibiotic administration, fever, watery diarrhea, and elevated CRP levels persisted after stoma closure. Escalation of systemic steroid therapy resulted in rapid improvement of clinical and laboratory parameters and was followed by the addition of oral mesalamine and subsequent tapering of PSL until discharge. CMZ, cefmetazole; CRP, C-reactive protein; MEPM, meropenem; PSL, prednisolone; TAZ/PIPC, tazobactam/piperacillin; WBC, white blood cell

**Fig. 8:**
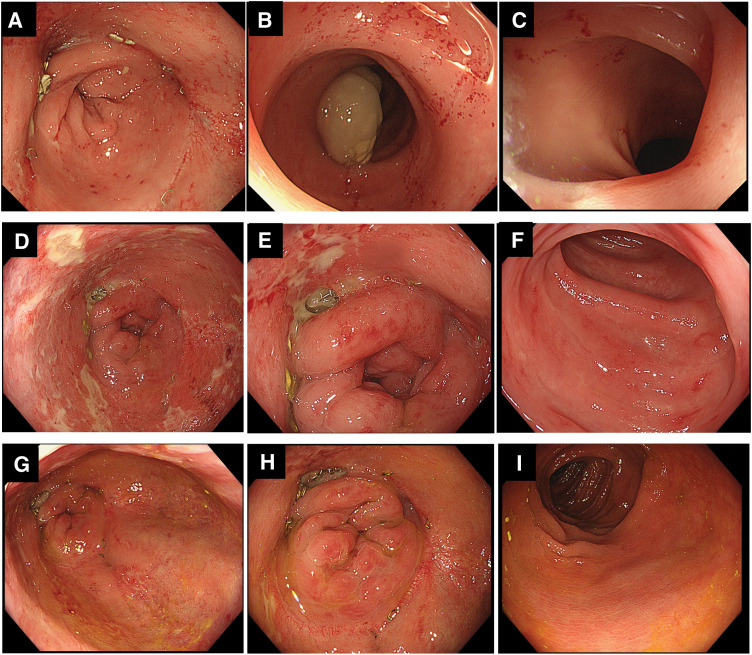
Colonoscopy findings before and after stoma closure. (**A**) Preoperative colonoscopy showing complete healing of the previous anastomotic leak. (**B**, **C**) No abnormal findings were observed in the proximal colon before stoma closure. (**D**, **E**) Colonoscopy on POD 7 demonstrating ulceration at the anastomotic site and erosive changes in the rectal mucosa. (**F**) Diffuse mucosal erythema, erosions, and edema in the proximal colon on POD 7. (**G**, **H**) Follow-up colonoscopy after escalation of steroid therapy showing marked improvement of the anastomotic ulcer and rectal erosions. (**I**) Resolution of inflammatory changes in the proximal colon.

The perioperative clinical course of stoma closure is summarized in **Fig. 7**. Preoperative colonoscopy confirmed complete healing of the previous anastomotic leak (**Fig. 8A**) with no abnormal findings in the proximal colon (**Fig. 8B** and **8C**). Stoma closure was performed with the patient on maintenance oral PSL of 1 mg/day. For perioperative steroid coverage, 50 mg hydrocortisone was administered intravenously on the day of surgery, and oral PSL at 1 mg/day was resumed the following day. Despite the absence of oral intake until POD 5, watery diarrhea and fever that did not respond to antibiotic therapy persisted. Laboratory tests revealed a white blood cell count of 8850/μL, which was within the normal range, whereas CRP levels increased to 11.51 mg/dL. Given the lack of response to antibiotic therapy and the discrepancy between the clinical findings and inflammatory markers, an exacerbation of inflammatory bowel disease rather than infection was suspected, and the PSL dose was increased to 10 mg/day. On the following day (POD 6), both fever and diarrhea resolved promptly. Colonoscopy performed on POD 7 revealed ulceration at the anastomotic site and erosive changes in the rectal mucosa (**Fig. 8D** and **8E**), along with mucosal erythema, erosions, and edema in the proximal colon (**Fig. 8F**). A diagnosis of UC exacerbation rather than diversion colitis was made, particularly because colitis recurred even after restoration of the fecal stream following stoma closure, which is inconsistent with diversion colitis, together with the compatible endoscopic and histopathological findings and the prompt response to systemic corticosteroid therapy. Histopathological examination demonstrated marked inflammatory cell infiltration in the mucosal stroma with cryptitis and crypt abscesses, consistent with findings from the previous hospitalization (**Fig. 6B**). The PSL dose was further increased to 30 mg/day, and oral mesalamine therapy at a dose of 4000 mg/day was initiated concurrently. This regimen was maintained for 10 days. After confirmation of improvement in inflammatory markers, follow-up colonoscopy showed marked improvement of the anastomotic ulcer and rectal erosions (**Fig. 8G** and **8H**), as well as resolution of inflammatory changes in the proximal colon (**Fig. 8I**). The PSL dose was subsequently tapered to 20 mg/day, and the patient was discharged on POD 20.

**Fig. 9:**
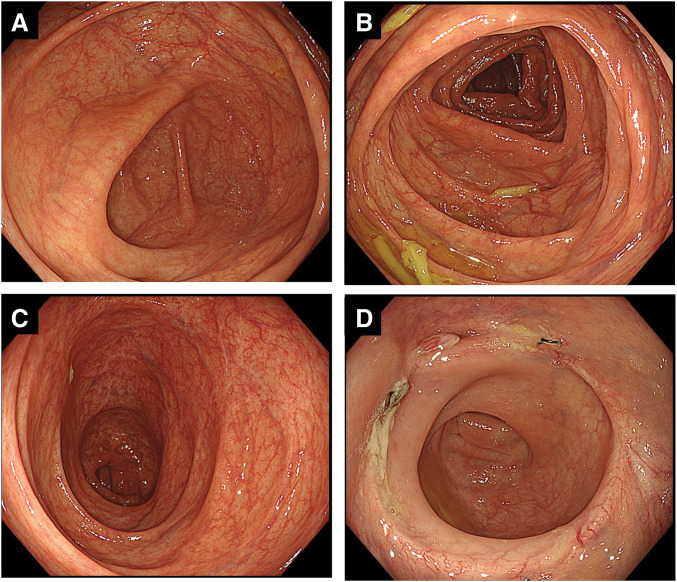
Surveillance colonoscopy findings at long-term follow-up. Surveillance colonoscopy performed more than 2 years after the initial surgery demonstrated normal colonic mucosa without ulceration, erosion, or active inflammatory changes. (**A**) Cecum. (**B**) Transverse colon. (**C**) Descending colon. (**D**) Anastomotic site, showing complete mucosal healing without evidence of recurrent inflammation or tumor.

Steroids were gradually tapered in the outpatient setting and discontinued several months later. At the most recent follow-up, more than 2 years after the initial surgery, the patient remains clinically well, maintained on mesalamine and probiotics without steroid use, with no recurrence of intestinal inflammation or colorectal cancer. Surveillance colonoscopy demonstrated normal mucosa without ulceration, erosion, or active inflammatory changes in the cecum (**Fig. 9A**), transverse colon (**Fig. 9B**), descending colon (**Fig. 9C**), or at the anastomotic site (**Fig. 9D**).

## DISCUSSION

The present case highlights several important clinical challenges in the postoperative management of colorectal cancer, particularly in distinguishing UC from diversion colitis in the setting of anastomotic complications. Development of UC after colorectal cancer surgery is extremely rare,^1,2)^ and postoperative inflammatory changes are typically attributed to diversion colitis, particularly in patients with a diverting stoma.^3,4)^ However, this case demonstrates that UC should be considered in the differential diagnosis when postoperative inflammation is refractory to conventional management or follows an atypical clinical course.

Diversion colitis is generally attributed to deprivation of short-chain fatty acids in the defunctionalized colon^4)^ and is observed histologically in most diverted colonic segments, although only a minority of patients develop clinical symptoms.^3)^ Endoscopic findings such as erythema, granularity, loss of vascular pattern, friability, and erosions are nonspecific and may overlap with those of UC, particularly in the postoperative period.^3,6,7)^ Consequently, differentiation based solely on endoscopic or histopathological findings is often challenging, and longitudinal assessment incorporating disease onset, clinical course, response to treatment, and recurrence is essential.^6)^ Although reports describing subtle preoperative inflammation as an early manifestation of UC are limited, nonspecific endoscopic findings such as erythema and friability may precede a definitive diagnosis and overlap with other inflammatory conditions. Furthermore, histopathologic evaluation alone is insufficient for accurate differentiation, underscoring the need for comprehensive longitudinal clinical assessment.^3,7)^

In the present case, the initial diagnosis of diversion colitis during the second hospitalization was reasonable given the timing and clinical context; however, the subsequent disease course prompted suspicion of an underlying inflammatory bowel disease rather than diversion colitis alone.

UC is often subject to substantial diagnostic delay owing to nonspecific symptoms, normal or non-diagnostic initial endoscopic findings, and misattribution to other conditions. Such delays, ranging from months to years, have important implications for disease progression and outcomes.^8)^ In this context, the delayed recognition of UC in the present case is consistent with previously reported diagnostic challenges rather than an exceptional circumstance.

A notable feature of this case was the occurrence of 3 anastomosis-related complications at different time points: anastomotic leak in the immediate postoperative period, deep ulceration during the diversion period prior to postoperative chemotherapy, and recurrent ulceration after stoma closure. Repeated involvement of the anastomotic site suggests increased inflammatory vulnerability of the colonic mucosa rather than purely mechanical or infectious etiologies. Importantly, all episodes demonstrated a marked response to systemic steroid therapy, which is atypical for diversion colitis alone and strongly supports an immune-mediated inflammatory process.

Several additional clinical features suggest that surgical stress and postoperative environmental changes played a significant role in triggering UC in this patient. First, anastomotic leak occurred unusually early, on POD 1, earlier than typically observed in conventional anastomotic failure. Second, despite the presence of a diverting stoma, high-grade fever and elevated inflammatory markers persisted for a prolonged period. Third, purulent drainage from the surgical drain continued for more than 2 months. These findings cannot be explained solely by mechanical failure or infection and instead suggest an exaggerated inflammatory response associated with immune dysregulation.

Although diagnosing UC at the time of the initial anastomotic leak would have been challenging, retrospective review revealed mild erosive changes in the cecum on preoperative colonoscopy. These findings were initially interpreted as nonspecific inflammation and were not further investigated; however, in retrospect, they may have represented an early manifestation of UC and an important overlooked clue. This observation emphasizes that even subtle inflammatory changes identified preoperatively in patients with colorectal cancer warrant careful evaluation, including histopathological assessment, particularly when postoperative complications follow an atypical course.

In general, patients with colorectal cancer complicated by established UC often undergo total colectomy, making clinical situations such as the present case uncommon.^9)^ Nevertheless, this case suggests that postoperative stress, alterations in gut microbiota, and disruption of mucosal immune homeostasis may collectively contribute to the onset or manifestation of underlying UC after colorectal cancer surgery. The relapse of inflammation after stoma closure is particularly noteworthy, as diversion colitis typically improves following restoration of intestinal continuity,^3,6)^ whereas recurrence strongly favors UC as a systemic immune-mediated disease rather than a localized reactive condition. Taken together, the recurrence of diffuse colitis after restoration of the fecal stream, the presence of cryptitis and crypt abscesses on histopathology, and the prompt response to systemic corticosteroid therapy supported an immune-mediated inflammatory process resembling UC rather than diversion colitis alone. In addition, recent reports suggest that diversion colitis itself may contribute to the progression or manifestation of underlying UC. Yaguchi et al. reported cases in which UC progressed following diversion colitis, supporting the concept that fecal diversion and subsequent mucosal immune alterations may influence the natural progression of UC.^10)^

Despite having stage IV colorectal cancer and experiencing 3 anastomosis-related complications, the patient ultimately achieved sustained, steroid-free remission of UC and has remained free from both intestinal inflammation and cancer recurrence on mesalamine monotherapy alone. Although this favorable outcome cannot be generalized, the clinical implications of this case are substantial, underscoring the importance of considering immune-mediated inflammation in patients with atypical postoperative courses and highlighting the need for heightened awareness of UC as a potential contributor to postoperative complications following colorectal cancer surgery.

## CONCLUSIONS

Development of UC after rectal cancer surgery is extremely rare but should be considered when postoperative colitis shows an atypical course or repeated anastomotic involvement. Although diversion colitis is common in diverted segments, persistent or recurrent inflammation that responds to systemic steroids may indicate immune-mediated disease rather than diversion-related changes alone. Thus, careful endoscopic evaluation and clinical assessment are essential for appropriate diagnosis and management. Although this observation is based on a single case, it provides clinically important insight into postoperative inflammatory complications following colorectal cancer surgery.
